# Platelet-rich Plasma for Skin Rejuvenation and Treatment of Actinic Elastosis in the Lower Eyelid Area

**DOI:** 10.7759/cureus.2999

**Published:** 2018-07-18

**Authors:** Matthias Aust, Hanno Pototschnig, Sarina Jamchi, Kay-Hendrik Busch

**Affiliations:** 1 Plastic Surgery, Waldkrankenhaus Bonn, Bonn, DEU; 2 Regenerative Medicine, Munich Medical Esthetic, Bonn, DEU

**Keywords:** prp, platelet-rich plasma, acp, autologous conditioned plasma, skin rejuvenation, actinic elastosis, lower eyelid, under-eye area, dark circles, personalized cell therapy

## Abstract

Background

Treatment of the lower eyelid region to rejuvenate the skin or treat actinic elastosis often proves difficult. Established treatment options, such as hyaluronic acid injections, botulinum toxin injections, microneedling, skin resurfacing (microdermabrasion, chemical peel (exfoliation), laser treatment), as well as blepharoplasties and autologous fat transfers, can be associated with significant risks and increased patient burden. Furthermore, they may not be effective for treating the signs of skin aging or actinic elastosis, including dark rings under the eyes, a lack of volume and cutis laxa. A minimally invasive treatment approach which visibly improves the above-mentioned conditions and which involves minimal risk and patient burden would be a desirable alternative.

Materials & methods

Twenty patients were treated a total of three times at monthly intervals with PRP (platelet-rich plasma). The patients were examined on the days of treatment and one month after the third injection. The PRP was obtained directly prior to treatment using the Arthrex ACP double syringe at the point of care. The injections (2 ml PRP per side) were administered laterally using 27 G 38 mm cannulas. Accurate photographic documentation and skin elasticity measurements using a cutometer were performed to objectify the subjective assessments from the patient and practitioner questionnaires.

Results

A progressive improvement in the esthetic outcome and a high level of patient satisfaction were determined. The cutometer measurements showed a statistically significant higher level of skin firmness (due to increased collagen production) and a statistically significant increase in skin elasticity (thanks to increased elastin production). Other than the anticipated visible swelling directly after the PRP injection, no other undesirable side effects or complications occurred. The typical burning sensation during the injection had not been reported.

Conclusion

The results indicate that a series of PRP injections in the lower eyelid region is a safe, efficient, virtually pain-free, simple and rapid treatment option for an area with otherwise limited treatment alternatives.

## Introduction

The eye region is located in the center of the human face. Studies of the visual field have shown that men first of all look women in the eyes. Women, on the other hand, initially look slightly below the eyes of men [1]. Therefore, the overall skin quality, especially of the face, plays a central role when assessing attractiveness.

The anatomy of the human face is complex. A particular feature of the lower eyelid region is that there is no superficial fatty layer to support the skin. This, on the other hand, is a major factor behind the early visibility of aging processes [2]. The particularly thin skin, the elasticity and the tone of the ligaments are also fundamental here.

Alongside the described chronological aging process which affects the skin, actinic elastosis is a further important aspect. Ultraviolet (UV) radiation results in the formation of reactive oxygen species, increased production of matrix metalloproteinases and induction of the transcription factor AP-1, which ultimately inhibits collagen production and causes accelerated collagen fiber collapse [3]. Moreover, UV radiation inhibits the expression of TGF-β2, which promotes collagen production [4]. Whilst this UV-related skin damage is initially not visible, over the course of time and subject to continued harmful UV exposure, wrinkles and actinic elastosis will ultimately result [5].

According to the definition of Marx, platelet-rich plasma (PRP) is autologous plasma with an increased platelet concentration, compared to the whole blood (baseline) [6]. PRP contains numerous growth factors that are responsible for its effectiveness. The growth factors are released following endogenous or exogenous activation of the platelets and then have a chemotactic effect and act directly and indirectly to regenerate the tissue. Some of the platelets are activated by mechanical influences during centrifugation. Collagen activates the platelets in vivo endogenously while needle-induced bleeding from the injection may additionally contribute to the clotting. Exogenous activation by means of adding calcium has become less popular in recent years [7].

Mesenchymal stem cells and fibroblasts are attracted by PRP. Their proliferation is stimulated, which can have a positive effect on skin quality [8-10]. Literature indicates that the ideal platelet concentration is 2.5 times over baseline; both higher and lower concentrations resulted in a less potent stimulation and proliferation of the fibroblasts [9]. In addition, with a platelet concentration in this range, the highest secretion of endogenous hyaluronic acid and type I procollagen was described for skin fibroblasts [11].

PRP with low erythrocytes and leukocytes levels seems advantageous for the majority of applications, since proinflammatory cytokines within the erythrocytes can result in the generation of free radicals. These can damage the skin, while high levels of leukocytes can cause negative effects due to the release of proteases [12-13]. In their review “Evaluating Platelet-Rich Therapy for Facial Aesthetics and Alopecia: A Critical Review of the Literature” the authors assume that the preparation of PRP influences the clinical results and demand prospective controlled trials to determine the method of PRP preparation and application most likely to produce optimal clinical results [14].

Loibl et al. showed that using the Arthrex ACP double syringe (Arthrex Inc, Naples, FL, USA), a PRP with approx. 2.5 times the normal platelet concentration can be produced which is practically free from erythrocytes and leukocytes [15]. The safety and efficiency of this system has been demonstrated in a Food and Drug Administration (FDA)-sanctioned Level-1-Study [16].

PRP has been used in dermatology, plastic surgery and aesthetic medicine, wound care, sports medicine, orthopedics, trauma surgery as well as oral surgery and dentistry for many years [16-22]. Possible therapeutic applications are mono or combination treatments of actinic elastosis, several forms of alopecia, (acne) scars, post-laser treatments, osteoarthritis, tendinopathies, tooth extractions, infrabony defects, tooth implants, jaw bone lesions as well as chronic wounds (diabetic leg ulcers). In the field of aesthetic medicine, PRP injections are popular for skin rejuvenation.

In numerous cases, established minimally invasive procedures (hyaluronic acid and botulinum toxin injections, microneedling, skin resurfacing (microdermabrasion, chemical peel (exfoliation), laser treatment) as well as blepharoplasties and autologous fat transfers), do not provide substantial skin textural improvement, fail to achieve the desired result, and are associated with the risk of complications. Also, many patients do not wish to undergo surgery therefore the aim was to evaluate PRP injections, a novel, simple and atraumatic treatment option that is able to counter both actinic elastosis and chronological aging processes.

## Materials and methods

Demographic data, number of patients, inclusion/exclusion criteria

This prospective study comprised 20 patients. Male and female patients aged between 21 and 60 with rings under their eyes, volume deficits and excess skin in the lower eyelid region were treated. All patients allowed the three planned injections to be administered. Sixteen of the patients were female, four male. The average age was 45. The table below (Table 1) shows the percentage distribution according to age groups, with the lowest age being 21 and the highest 60.

**Table 1:** Distribution of age groups.

Age	
20-29	15%
30-39	10%
40-49	30%
50-59	35%
60	10%

Patients who had undergone previous surgery in the eyelid region, previous minimally invasive treatments such as botulinum toxin or fillers in the eyelid region as well as patients with platelet functional disorders (blood diseases), active skin disorders or inflammations, acute or chronic infections, tumors or cancer were excluded from this study, as were patients undergoing anticoagulant treatments and smokers.

Documentation and measurement method

Before being admitted to the study, the patients were checked to ensure compliance with the inclusion/exclusion criteria, and their demographic data was recorded.

The patients were photographed (Canon EOS 1200D; Canon K.K., Tokyo, Japan) at T1 (prior to first injection), T2 (prior to second injection), T3 (prior to third injection) and T4 (one month after the third injection). Objective skin measurements were also taken at these times using the cutometer MPA 580 (COURAGE+KHAZAKA electronic GmbH, Cologne, Germany). The R0 values were measured to determine skin firmness, whereby lower values indicate a higher firmness, and F0 and F1 were measured to determine skin elasticity, whereby lower values mean higher elasticity as per the manufacturer’s instructions. The patients also underwent a clinical examination, and questionnaires with items with closed answer options relating to Quality of Life, Patient Satisfaction, and Physician’s and Patient’s Global Assessment Grades were completed by the subjects at T2, T3 and T4. Thus, in addition to recording patient satisfaction and perception, volume increase, increase in elasticity/firmness of skin, reduction in rings under the eyes were documented. By using the Physician’s and Patient’s Global Assessment Grades, it was possible to record the subjective assessments of the treating physician and another physician, as well as the patients’ assessments.

Preparation of PRP and injection technique

**Figure 1:**
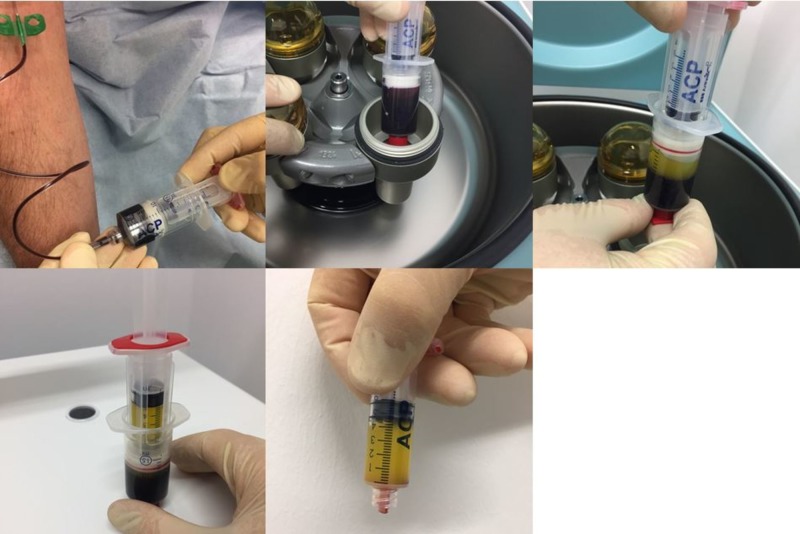
Preparation of platelet-rich plasma (PRP).

Three treatments with PRP were performed at monthly intervals on each patient. During each treatment, 15 ml of blood was taken from the patient via a butterfly into an Arthrex ACP double syringe. Directly after this, the syringe was centrifuged horizontally in a Hettich Rotofix 32 A centrifuge (Andreas Hettich GmbH & Co.KG, Tuttlingen, Germany) for five minutes at 1500 rpm (= 350 G). The PRP settled in the upper third of the syringe (approx. 5 to 6 ml) and was drawn into the inner syringe (Figure 1). The inner syringe was twisted out and, following local disinfection, 2 ml of PRP (per side) was injected intradermally/subdermally in the patient's lower eyelid region using 27G 38 mm needles. This was followed by approximately five minutes of localized cooling with a cooling bag and compression with Steristrips.

Statistical evaluation

The objective measurement parameters (cutometer measurements) were evaluated statistically using Welch’s t-test; the significance level was set at p =< 0.05.

## Results

Clinical findings before and directly after the treatment/side effects

The indications were rings under the eyes (eight patients), lack of volume (17 patients) and excess skin (nine patients). Swelling occurred one to three days after treatment together with a slight feeling of pressure as a result of the injected volume. This was alleviated by cooling and taping. Discrete hematomas were experienced by a minority of patients, as minor veins were injured by the injection. No serious side effects occurred.

Questionnaires

The patients were either satisfied or very satisfied with the perceived changes in the lower eyelid region, and continuously improved from injection to injection. Family, friends and work colleagues also regarded the results as very positive. The patients are willing to continue and recommend the treatment.

In terms of the Physician’s and Patient’s Global Assessment, the data indicated a gradual improvement/increase in the skin volume/skin thickness and skin elasticity as well as a reduction in excess skin, wrinkles and rings under the eyes. The assessment given by the physicians barely deviated from that of the patients.

Cutometer measurements

The cutometer measurements showed significantly lowered values for R0 (Table 2), F0 (Table 3), F1 (Table 4). This infers greater skin firmness (due to increased collagen production) and increased elasticity (due to greater elastin production).

**Table 2:** Reduction of R0 values. The R0 values dropped steadily. The value for increased skin firmness is statistically significant.

		T1	T2	T3	T4	Welch’s t-test T1/T4	
Mean	R0	0.34573684	0.2646	0.1814	0.16565		
Standard deviation	R0	0.17541691	0.1373859	0.082681	0.09860622		
Number of patients	R0	19	20	20	20		
						p-value	0.000514304

**Table 3:** Reduction of F0 values. The F0 values dropped steadily. The value for increased skin elasticity is statistically significant.

		T1	T2	T3	T4	Welch’s t-test T1/T4	
Mean	F0	0.08632105	0.06296	0.05417	0.04425		
Standard deviation	F0	0.04325455	0.0338776	0.02866878	0.03509496		
Number of patients	F0	19	20	20	20		
						p-value	0.00212529

**Table 4:** Reduction of F1 values. The F1 values dropped steadily. The value for increased skin elasticity is statistically significant.

		T1	T2	T3	T4	Welch’s t-test T1/T4	
Mean	F1	0.05310526	0.040505	0.032385	0.024905		
Standard deviation	F1	0.03392506	0.0281275	0.02577917	0.01904649		
Number of patients	F1	19	20	20	20		
						p-value	0.00359472

Clinical results (photographic documentation)

**Figure 2:**
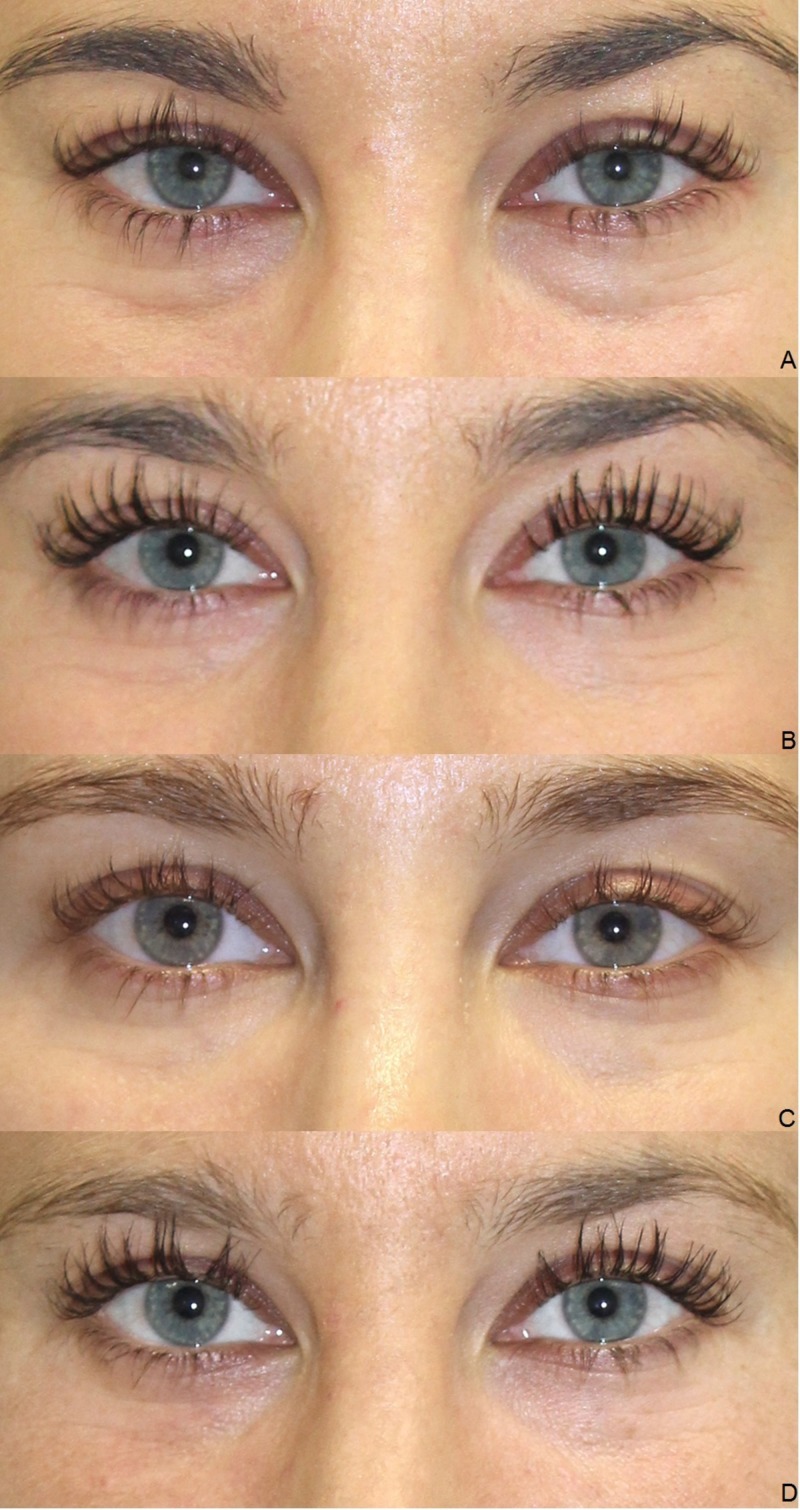
Treatment results. Female, 23 years, before the first (A), before the second (B), before the third (C), and four weeks after the third injection of platelet-rich plasma (PRP) (D).

**Figure 3:**
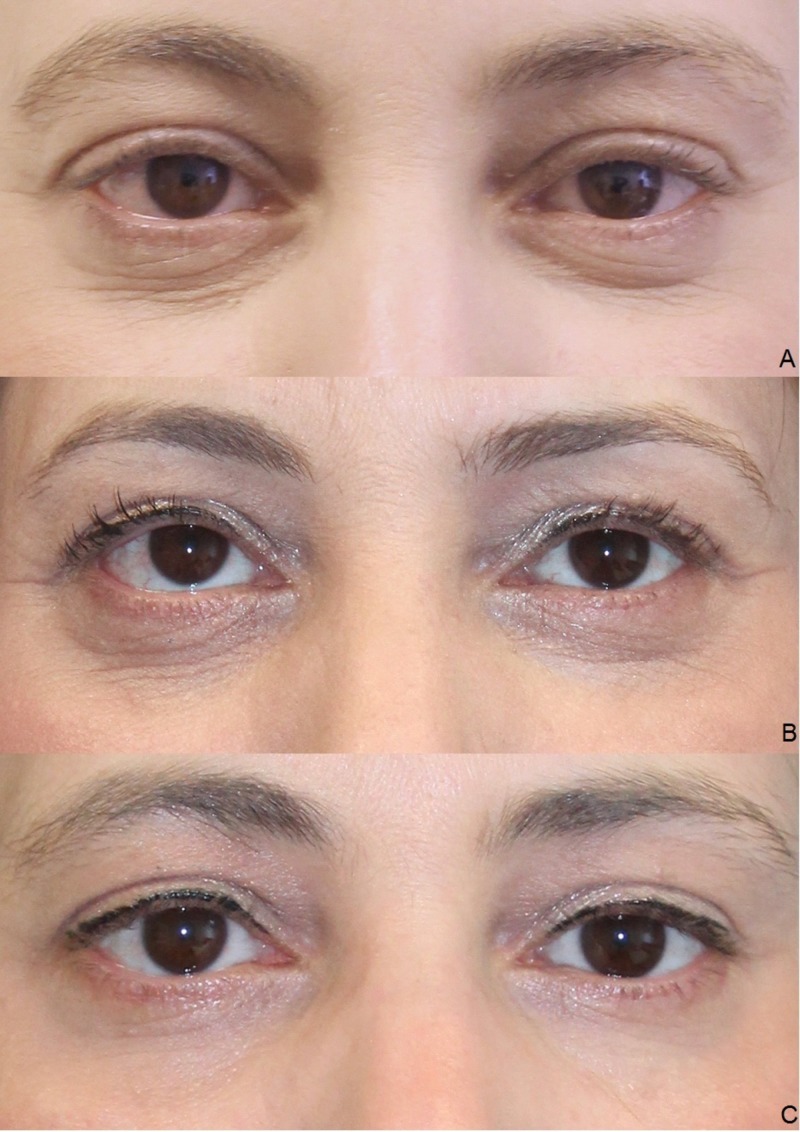
Treatment results. Female, 42 years, before the first (A), before the second (B), and before the third injection of platelet-rich plasma (PRP) (C).

**Figure 4:**
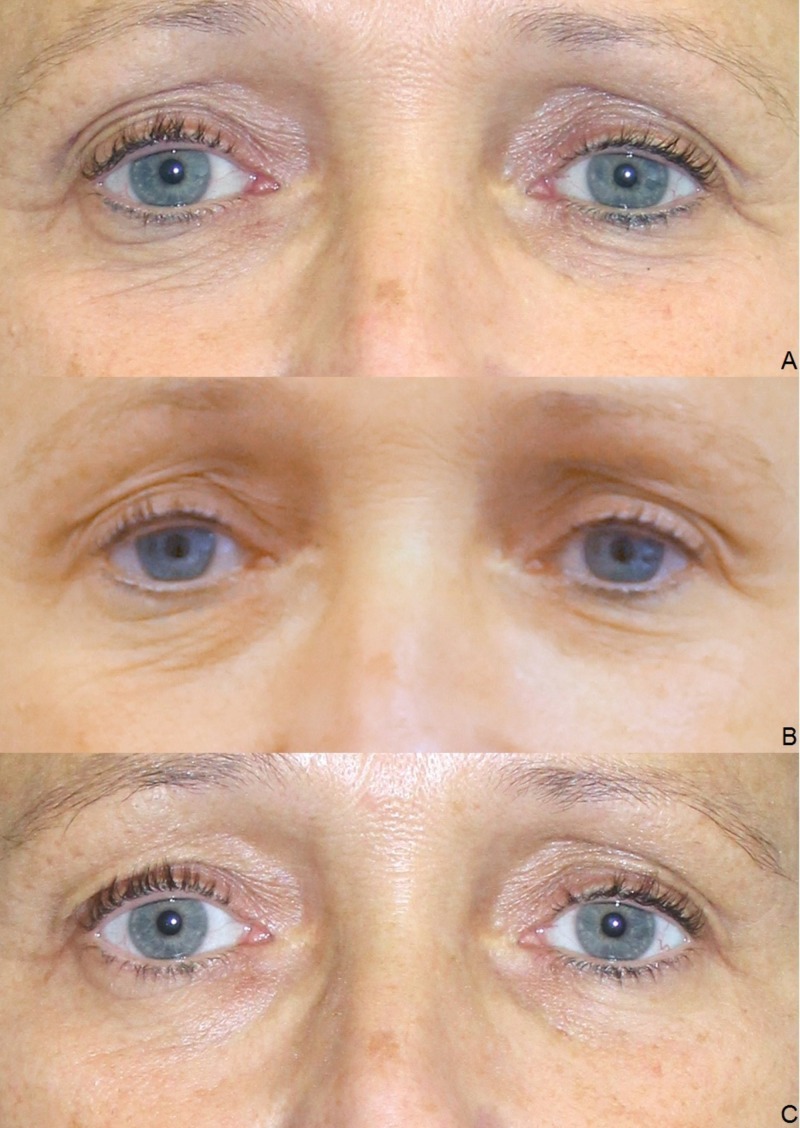
Treatment results. Female, 54 years, before the second (A), before the third (B), and four weeks after the third injection of platelet-rich plasma (PRP) (C).

**Figure 5:**
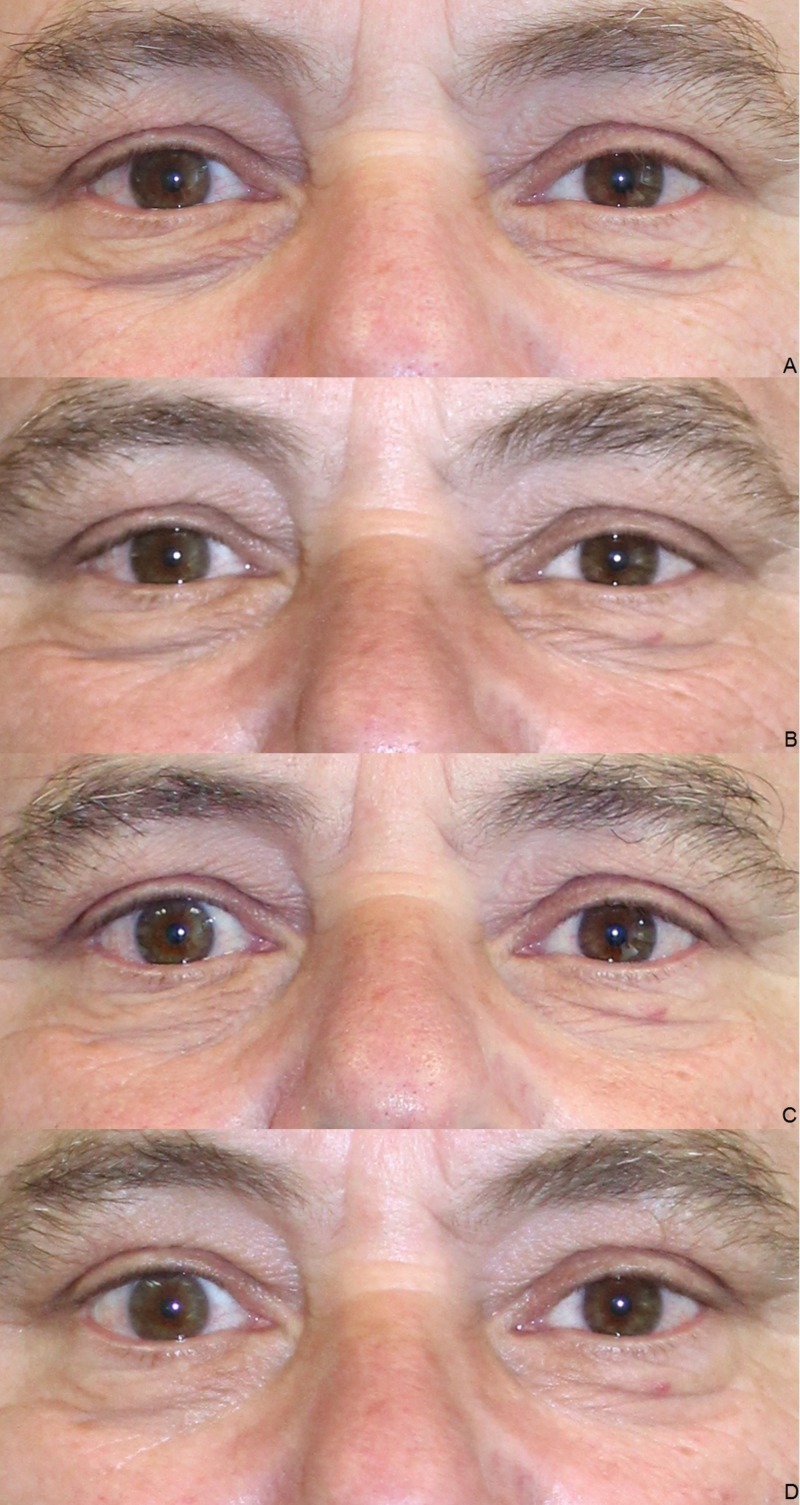
Treatment results. Male, 60 years, before the first (A), before the second (B), before the third (C), and four weeks after the third injection of platelet-rich plasma (PRP) (D).

**Figure 6:**
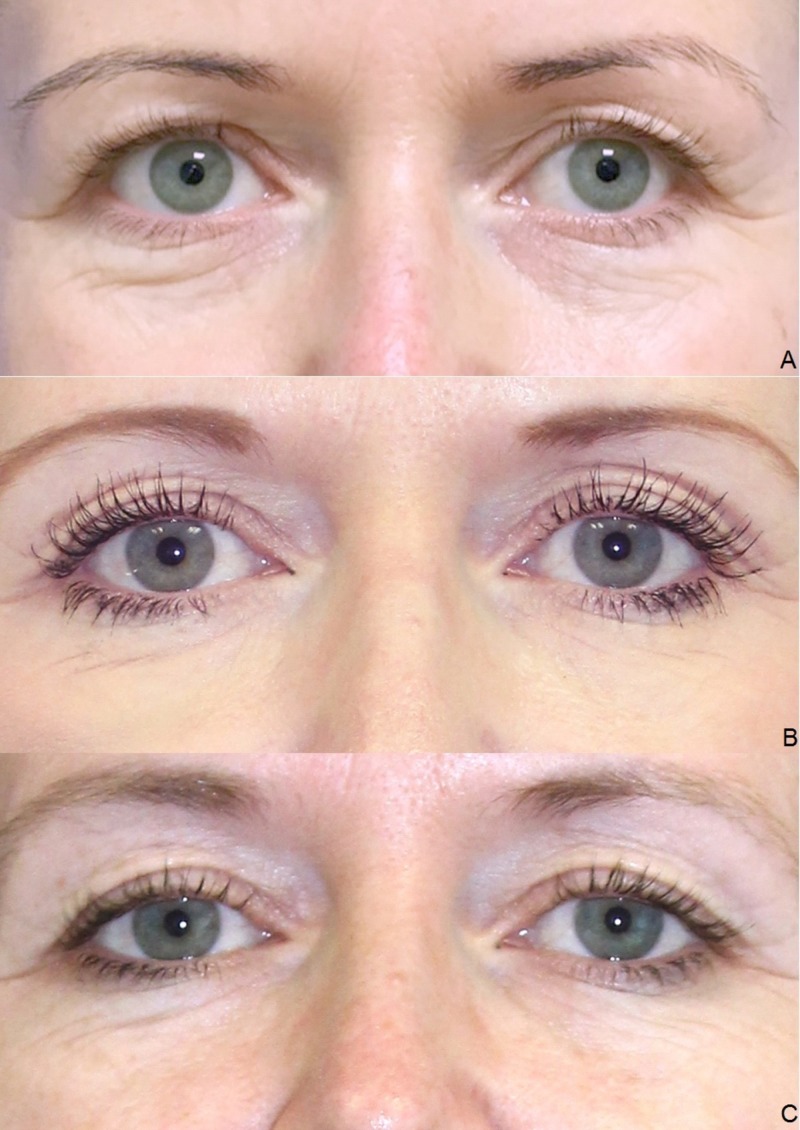
Treatment results. Female, 38 years, before the first (A), before the third (B) and four weeks after the third injection of platelet-rich plasma (PRP) (C).

The clinical examination showed a gradual initiation of the effect. The overall results were satisfactory after the end of the treatment series, irrespective of age and gender (Figures 2-6).

## Discussion

With its very thin skin and complex anatomy, the lower eyelid region is an extremely difficult area to treat. Not only processes of chronologic aging but also actinic elastosis lead to symptoms such as dark rings under the eyes (caused by thin skin and the visibility of veins and/or the orbicularis oculi muscle through this skin), volume deficits and excess skin. The established minimally invasive treatment methods and surgical procedures often fail to achieve the desired results and are sometimes risky. In the past, hyaluronic acid fillers were only injected intradermally and were thus superficial. Today, these are also used to augment deeper subcutaneous regions, which can help to remodel the anatomic structures. Hyaluronic acid fillers can be placed supraperiosteally, using a vertical or horizontal technique [2].

The use of fillers demands a high level of expertise of the physician, and can potentially make the symptoms even worse. Given the thinness of the skin, all color irregularities and low-grade inflammations are particularly visible. Moreover, this region is predisposed to swelling, which can cause problems with hydrophilic hyaluronic acid fillers in particular. In the area of the lacrimal groove and the lower eyelid region, the Tyndall effect may result in bluish discoloration. Dark rings due to a caudal cheek fat deposit cannot be treated with hyaluronic acid, since a SOOF projection leads to swelling of the fat chamber and an unnatural overprojection [2]. Goldberg and Fiaschetti also describe considerable complication rates of 11% from contour irregularities, 10% from hematomas, 9% from color changes and 15% from fluid retention. They used hyaluronidase after 15% of all injections to perform corrections [23]. Botulinum toxin injections are suitable for treating lateral canthal wrinkles (crow's feet), as these result from contractions of the orbicularis oculi muscle. It takes around two weeks for the effects of botulinum toxin to become fully visible and these then last around three to four months. The formation of an ectropion should be mentioned here as a possible complication. Since botulinum toxin merely serves to inhibit local muscle contraction, but does not have a regenerative effect on the surrounding tissue and skin, the results of the treatment on aging skin in the lower eyelid region are mostly not satisfactory.

Due to the extremely thin skin and vascularization in the treatment area, microneedling is associated with a high risk of hematomas. Furthermore, it is painful and thus not a popular treatment choice for this region. The restricted accessibility to the eyelash line also makes treatment more difficult. Moreover, with microneedling, only the skin quality is improved, but this does not offer an accurate procedure for treating volume losses. Skin resurfacing methods are mostly used as combination and not as stand-alone treatment. Again, only the skin quality is improved without any effect on volume loss. As for laser treatments, the risks of pigmentation and scarring must be considered. Lower lid blepharoplasties are a successful surgical treatment method, yet risks such as lower lid malposition, excessive display of the sclerae, round lid slits and infraorbital emptiness are not inconsiderable. Alongside blepharoplasties, autologous fat transfers are also successfully used in the surgery to treat the lower eyelid region. Particular care must be taken with patients with very thin skin, as any irregularities following insertion may remain visible. There is also the risk of asymmetries from the uneven resorption of fat.

Given that some minimally invasive procedures often achieve unsatisfactory results with potential side effects, the question needs to be asked as to whether the injection of PRP is an alternative treatment approach. The stimulative effect of PRP on fibroblasts was demonstrated years ago by in vitro studies [9]. PRP stimulates the secretion of hyaluronic acid and type 1 procollagen in skin fibroblasts [11]. A number of clinical studies have been published describing the positive effects of PRP on the appearance of the skin. In their recently published review, Motosko et al. reported improved skin texture, color homogeneity, firmness and elasticity, increased volume, dermal thickness and patient satisfaction, reduced solar elastosis, decreased wrinkles, severity of nasolabial folds, acne scars, erythema and melanin after solely facial application of PRP [14]. Within their systematic review, they stated, that the majority of studies support PRP as a beneficial treatment in facial aesthetics. PRP appears to be safe, with a low-risk profile. Although there is a theoretical risk of injecting high-density platelet solution into a vessel, there were no cases of such complication observed in this literature search. Therefore, although platelet-rich plasma may not be the panacea of facial rejuvenation, there exists a place for this treatment in plastic surgery [14]. Apart from its solely application PRP is also frequently used as a supportive therapy [17]. In the PRP group of their randomized study, “Platelet-Rich Plasma Combined with Fractional Laser Therapy for Skin Rejuvenation”, Shin et al. describe a higher level of subjective satisfaction in terms of skin elasticity, which was confirmed by objective cutometer measurements [24]. They further concluded that the proliferation of keratinocytes and fibroblasts and the production of collagen may explain why PRP is able to improve dermal elasticity. In a placebo-controlled study, Abuaf et al. performed histological examinations. In the PRP group, the histological results were statistically significantly better than in the control group. The average optical density of collagen improved by 89.05% in the PRP group. On the control side, the density improved by 46.01%, which can be attributed to the needling effect from the injection [25].

The following five studies have been published on the treatment of the lower eyelid region with PRP [26-30]. In the study by Mehryan et al., eight out of ten patients (80%) achieved fair to good improvements after three months. On a scale from 0-3, the mean value was 1.7. Ninety percent of patients assessed their treatment results as either excellent or good. In terms of satisfaction, the mean value was 2.2 on a scale of 0-3. All patients reported a burning sensation upon injection. Minor temporary dermatorrhagias were the only side effect. Persistent or serious side effects were not described [26].

In their study, Banihashemi et al. treated 30 subjects with PRP in two sessions at three-month intervals. At the three- and six-month follow-up, the majority of patients reported fair to excellent results in terms of dark rings under the eyes, periorbital wrinkles, nasolabial folds and skin firmness. Seventeen percent of patients reported only minor treatment success or none at all. The treatment success was viewed differently by patients, the treating physician and a second independent physician. The best effects were seen in the reduction of dark rings under the eyes and wrinkles [27].

Al-Shami conducted a study with 50 patients on the treatment of periorbital hyperpigmentation with three PRP injections at monthly intervals. Four percent of patients described excellent results, 12% significant results, 46% fair results and 38% poor results. As per the medical assessment, approx. 60% of patients showed fair to significant improvements. More than 65% of patients were either satisfied or very satisfied with the end results [28].

Kang et al. evaluated 16 patients in their study. 12.5% were extremely satisfied with the improvement in infraorbital wrinkles, 25% were very satisfied and 56.3% satisfied. The patients rated the improvement of infraorbital tone with 37.5% as very satisfied and 62.5% satisfied. In the blind assessment by three reviewers, the treatment results were evaluated as follows: 12.5% of the cases were rated as good, 18.8% as fair, 56.3% as poor, and in 12.5% of cases no improvement at all was seen. The erythema index dropped from 8.52 to 7.37 after PRP treatment [29].

Cameli et al. demonstrated an improvement in elasticity (measured with cutometer) in their study of 12 patients. Just as in our study, treatment involved three sessions at monthly intervals. The canthal region, forehead, cheeks and the nasolabial region were treated. In terms of skin texture and fine wrinkles, from a medical perspective the treatment results were assessed as good in 37.5% of cases, sufficient in 37.5% of cases and insufficient in 25% of cases. The patients gave the following assessments: 25% good, 37.5% sufficient, 37.5% insufficient [30].

Our study results are consistent with those reported in literature [26-30]. The average patient age in our study was 45; around two-thirds of patients were between 40 and 59 years of age. It is noteworthy that 15% of patients were in their early twenties, which indicates that the lower eyelid region can be affected by signs of ageing even in younger years. Especially for this demographic, it is important to have a simple and cost-efficient treatment option in addition to the costly surgical procedures. No relevant differences could be determined between the age groups in terms of the results. The positive treatment response, including in the over 60 age group, is striking, considering that the body’s ability to regenerate declines with increasing age. Thanks to objective measurement methods (cutometer), we were able to confirm our positive subjective data and clinical photographic results. Accurate documentation is not only for study purposes but also in daily practice important as the effects arise gradually. Assessment of skin improvement is challenging. The standardization of photographic documentation is vital and with the cutometer one more modality for assessment of the treatment effects has been found. 3D scans could be additionally a suitable tool for precise evaluation but their application is currently limited due to the high costs. Concerning the cutometer measurements the R0 value, which is an indicator of skin firmness and, which can be used indirectly to draw conclusions as to collagen fiber production, dropped steadily from 0.34573684 to 0.16565. At a p-value of p < 0.000514304, this positive value is statistically very significant. The F0 value, which is an indicator of skin elasticity, and can be used indirectly to draw conclusions as to elastin fiber production, dropped steadily from 0.08632105 to 0.04425. At a p-value of p < 0.00212529, this positive value is statistically very significant. This result is also confirmed by the likewise statistically very significant drop in the F1 value from 0.05310526 to 0.01904649 (p < 0.00359472). The last follow-up was one month after the third injection of PRP. We are planning further studies to evaluate the long-term efficiency of this treatment modality.

Quality of life in all areas was assessed as very positive following the conclusion of treatment. The patients were either satisfied or very satisfied with the positive changes in the lower eyelid region. The eyelid region is also seen as more youthful than before treatment. Those close to the patients also perceived continuous treatment successes. As such, it is hardly surprising that the patients were very willing to undergo further injections and recommend this treatment to others. It is striking that there is no mention of the symptoms being made worse in any of the Physician’s and Patient’s Global Assessment items, in either the medical or patient assessment; this shows just how safe this 100% autologous personalized cell therapy is. The steady increase in mean values for all items confirms the findings from the photographic documentation and is in keeping with the cutometer measurements. In terms of skin thickness, different degrees of improvements were recorded for all the patients. A similarly positive picture was also seen for skin elasticity. In terms of excess skin, the majority of patients saw improvements to differing extents. The PRP was injected both intradermally and subdermally also in proximity to the ligaments. It is assumed that the reduction in excess skin is achieved by the improvement of ligament tone and the increased volume most likely due to proliferation of tissue in the subdermal area. There was a continuous reduction in wrinkles with almost all patients noticing positive effects overall. In our study, there was a slight improvement in the rings under the eyes in the majority of patients. Signs of both chronologic ageing as well as actinic elastosis improved.

PRP can be produced and injected simply and quickly. The growth factors contained in the PRP have positive influences on the chronological aging processes as well as actinic elastosis. The effectiveness of this treatment can be explained by the stimulation of fibroblasts, increased production/secretion of collagen and endogenous hyaluronic acid, and recovery of ligament elasticity. No serious side effects occurred other than short-term swelling due to the injection volume, small-scale temporary bleeding and minor discomfort. This is a major advantage compared to the established injection therapies and surgical procedures. The PRP injections were well tolerated, the often occurring burning sensation when injecting PRP, which is documented in other studies [26, 30], was not reported by patients in our study. This is apparently due to the fact that the PRP contained no anticoagulants, which have an unfavorable pH value.

## Conclusions

Intradermal/subdermal injections of PRP obtained, using the Arthrex ACP double syringe, are a safe and efficient treatment option to achieve skin rejuvenation as well as to treat actinic elastosis. Significant treatment results are visible and measurable, patient satisfaction is high, and no serious adverse effects occurred. The minimum required volume of PRP should be quantified in further studies in order to render the treatment even more comfortable.
